# SCMR President's Page

**DOI:** 10.1186/1532-429X-13-47

**Published:** 2011-09-13

**Authors:** Scott D Flamm

**Affiliations:** 1 Cardiovascular Imaging Laboratory, Imaging Institute and Heart & Vascular Institute, Cleveland Clinic, Cleveland, Ohio 44195, USA

## Abstract

News from the Society for Cardiovascular Magnetic Resonance

## Editorial

The Society has begun 2011 on multiple highpoints, yet these have been tempered by a recent and particularly saddening low - the untimely death of Stefan Fischer. For those of you unaware, Stefan was the Director of Clinical Science for Philips Healthcare, North America, an important developer of CMR pulse sequences, tools and equipment that remain critical standards today; he was a long-standing member of SCMR with friends too numerous to count in our Society and field. I had known Stefan for well over a decade, and knew him as fiercely intelligent, strong-willed yet gently firm in his guidance, purposeful in increasing the benefits we impart to patients with our technology, and always practical. His passing leaves a gap that is impossible to fill, both in our Society, and in our hearts. I trust that the work we pursue in this Society will continue to live up to those characteristics and ideals that he embodied.

To recap key Society events thus far, the year began with the 2011 SCMR Annual Scientific Sessions held in conjunction with the Euro CMR Working Group in Nice, France. It was a great success with a record number of participants from around the world. Much of that success can be attributed to the constant and hard work put forth by the Program and Abstract Chairs, Sven Plein, MD, and Raymond Kwong, MD, who put together an outstanding scientific program with a compelling array of scientific presentations, excellent posters, invited talks, and thought provoking case-sessions. The opening plenary was an important prelude to the thrust of the meeting where Michael Lauer, MD, of the NHLBI, emphasized the critical importance of CMR investigators and clinicians to design trials that alter patient outcome, as opposed to simply providing a superior diagnosis - a theme that was emphasized repeatedly as necessary to maximize CMR's impact on clinical care.

During the meeting, the Society awarded its first Gold Medal Award, given for distinguished and extraordinary service to the field of Cardiovascular Magnetic Resonance and to the Society. Fittingly, Gerald Pohost, MD, and Charles Higgins, MD, two long-standing and esteemed leaders in CMR and the Society were the inaugural winners of this annual award.

Outside of the annual meeting fantastic news was received about our journal: the Journal of Cardiovascular Magnetic Resonance saw its impact factor increase from 2.28 to 4.33!, placing it within the upper tiers of both cardiovascular and imaging journals. This near doubling reflects the committed leadership of Journal Editor-in-Chief, Dudley Pennell, MD, to continually increase both the quality and quantity of CMR scientific publications, and to expand the reach and impact of the JCMR within the field of cardiovascular investigation. Great thanks are to be extended to Dr. Pennell and to the journal's Editorial Board for their tireless efforts on behalf of the JCMR. Please continue to send your important scientific work to our flagship journal.

Elsewhere, the Society continues to make progress in standardization of CMR, aspects that are essential for its continued adoption. In recent years the publication of recommendations for standardization of data acquisition [1] and reporting [2] were seen as important efforts for the use of CMR in the clinical arena, as well as amplifying the role of CMR in clinical trials. In 2011, under the direction of Jeanette Schulz-Menger, MD, we expect to see another important document for standardization of data post-processing.

Victor Ferrari, MD, the prior Education Committee Chair, along with James Moon, MD, the Web Editor, and his team have recorded and posted the majority of presentations from the 2011 meeting, making them available for continued review. In addition, the team has recently completed and made available on the Society's website, the first SCMR Online Course in CMR covering topics including CMR Physics, Safety, Techniques, and up to 35 Clinical Cases. These new educational courses are available for credit toward Level 1, 2, and 3 training in CMR, and should help facilitate training for those new to CMR. To further advance the Society's educational goals, SCMR has joined with the Euro CMR Working Group to develop an international standard credentialing examination for CMR to serve the needs of CMR practitioners world-wide. The exam will be available by the 2013 Annual meeting.

Moving now beyond recent accomplishments and looking to the immediate future, the Society's Board of Trustees has spent multiple days of intensive meetings in Strategic Planning that have led to refining the Society's Vision and Mission statements, along with identifying a series of themes - Societal Alliances & Partnerships, Clinical Evidence, Education, Communication, and Technological & Efficiency Advances - from which many new projects and directives have been developed. These Strategic Planning sessions, held at this year's annual meeting and at the mid-year Board meeting in Philadelphia in June of this year, have given us a roadmap of where we would like to go and how we can get there, as well as the tools for doing so more effectively. Expect that these projects will engage the Board, committees, and many of our members to help drive the numerous new initiatives.

As an international Society we have for years embraced a more global perspective, though one that remained relatively U.S., Europe, and Japan-centric; nonetheless, the rapidity and breadth of globalization necessarily requires a further expansion of our vision and ability to adapt. To date we are doing an admirable job: as highlights, a Middle East working group has been formed and a new imaging society in India seeks opportunities to collaborate; increasing activity from our Latin American group has led them to request that the Society consider holding an annual meeting in Latin America (not a question of if, but how soon); and through the efforts of former President Gerald Pohost, MD, we are making plans for a jointly sponsored meeting in China in the fall of this year, along with the development of a Chinese working group. To come full circle, the European Chapter (via the Euro CMR Working Group of the European Society of Cardiology) has developed an outstanding CMR registry that already has resulted in multiple publications; there are further ongoing efforts to expand the registry with help from the U.S. and Asia study groups, pushing it further toward a truly global CMR registry.

The Society is also expanding its outreach efforts and has recently nominated an SCMR member to a U.S. governmental advisory panel for imaging reimbursements, and in the process, and for the first time, collaborated with and cross-sponsored candidates from sister imaging societies (NASCI, SCCT, ISMRM, etc.). We expect more such policy-influencing nominations to come. Perhaps most importantly the Society is in process of a broad collaborative effort bringing together vendor partners, contrast manufacturers, the NIH and FDA, corelab and post-processing experts, and CMR thought leaders for design and initiation of a large-scale, multi-center clinical trial to push CMR to the forefront by demonstrating its clinical relevance and superiority - efforts that bring to reality the themes Michael Lauer, MD, emphasized at the 2011 annual meeting earlier in this report.

So in summary, I believe that this is an exciting and invigorating period for the Society, where the coming years will see growth along multiple avenues, continuing robustness and preference for CMR in cardiovascular care, and greater collaborative efforts that will further advance the field and the Society. The Board is working hard, and despite the challenging times we face today, the future continues to look bright.

To conclude, let me add that we are finalizing plans for the 2012 SCMR meeting. Raymond Kwong, MD, and Jeanette Schulz-Menger, MD, our Program and Abstract Chairs, respectively, have put together a terrific program. Multiple tracks aimed at clinicians, basic scientists, and pediatric CMR imagers are planned. Immediately prior to the annual meeting SCMR will hold a two-day joint workshop in concert with ISMRM: "Exploring New Dimensions of Cardiovascular Flow and Motion". We expect that this will be another spectacular annual meeting, and one you won't want to miss. Please remember the abstract deadline of October 3rd, and make your plans to come to Orlando, Florida, during February 2-5, 2012, where we hope to welcome many familiar and even more new faces to the meeting. For those making plans further ahead, be sure to pencil in your calendars the dates for the 2013 meeting to be held February 1-4, 2013 in San Francisco, California.
